# History of the Origin, and Transactions of the Medical Societies of Buffalo

**Published:** 1861-10

**Authors:** Thomas F. Rochester


BUFFALO

### Medical and Surgical Journal

AND REPORTER.

VOL. I. OCTOBER, 1861. NO. 3.

### ORIGINAL COMMUNICATIONS.

ART. I.—*History of the Origin, and Transactions of the Medical Societies of Buffalo, by* THOMAS F. ROCHESTER, M. D.

(CONTINUED.)

The preceding paper carries us to October 2d, 1855. At this meeting Dr. Hamilton presented a case of bent and partly fractured bone, in the clavicle of a child eleven weeks old. The peculiarity to which attention was invited, consisted in the fact that "*the bone, after being bent and partially broken, had immediately resumed its form.*" Dr. Hamilton then proceeded to explain what he considered the peculiar pathology of this class of accidents.

Dr. White next spoke of the microscopic diagnosis of Uterine affections, setting a high estimate upon the information afforded by examination of the exudations in doubtful cases, and illustrated his position by citing at length four instances, which terminated, under his care, in recovery. They had all been pronounced cancerous; but as the microscope did not reveal anything of a malignant nature, he was induced to resort to general and topical treatment, with the happy result mentioned.

At the November meeting, Dr. Rochester exhibited an enormous heart, taken from the body of a male patient, seventy years of age, who was brought to the Hospital in a dying condition. The heart, when washed and thoroughly cleansed, weighed forty-six ounces avordupois. It is one of the largest on record.

We find the next items of interest presented at the February meeting, 1856.

Dr. Hawley reported a case of rupture of the uterus, necessitating instrumental deliverance of the fœtus, terminating in the recovery of the mother.

Dr. White then gave, in detail, an account of a case of inverted uterus, restored by him one week after the accident. The patient died two days after the replacement. A post mortem examination, carefully conducted by Prof. Hunt, discovered no uterine laceration or peritonitis, and death was attributed to exhaustion from hœmorrhage, which had been alarming up to the time of the reposition of the organ.

Dr. Rochester then reported a second case of attempted suicide by strychnine. As in the former instance, camphor was employed successfully as an antidote. The patient, a muscular man, thirty two years of age, stated the amount of strychnine swallowed to have been four grains.

Dr. Baker, from the Committee on Organization, reported a form for an Act of Incorporation. This was ratified on the first day of April, 1856, and the "*Buffalo Medical Association*" became from that day an incorporated Society under the name and title of "*The Buffalo Medical and Surgical Association.*"

An election of officers was then held, and resulted in the choice of: Dr. Sanford Eastman, President; Dr. Austin Flint, Vice-President; Dr. Sanford B. Hunt, Secretary; Dr. James M. Newman, Treasurer; Dr. Wm. Howell, Librarian.

*June* 3*d*, 1856.—Dr. Flint read an able and elaborate paper on the "*Diagnostic Value of the Buffy Coat.*" He considered "the circumstances involved in its production," and the "significance and value belonging to it in its pathological relations, and the therapeutical indications furnished thereby." This paper is published in the twelfth vol. of the Buffalo Medical Journal, and is eminently worthy of perusal, especially by those who regard an excess of fibrin in the blood, as a *cause* of inflammation, and who seek to diminish the fibrinous element by resource to venœsection.

*July* 2d, 1856.—Prof. Hadley read, by appointment a report on Hair Dyes. He stated that "all the various dyes are made from the salts of two metals, viz., lead and silver—those of the latter are most extensively employed. "The quantity of silver thus subtracted from the currency is very large." "One firm in this city used last year 1,100 ounces of silver coin." Referring to Depilatories, Prof. Hadley said that all that he had examined contained arsenic, and were of course injurious.

*August* 5, 1856.—Prof. Hamilton presented a specimen of the middle finger, which had been torn off by machinery. Its peculiar feature was, that in being torn out, it removed with it one of the flexor tendons to the length of about fifteen inches. On the fourth day there was slight tenderness along the track of the tendon, but this immediately subsided. Prof. Hamilton considered that this wound healed thus kindly, on account of the entire exclusion of air.

*October 7th,* 1856.—Prof. Hamilton reported the results of two operations on congenital multilocular cysts. One terminated fatally from hœmorrhage; the other resulted in recovery, but was attended with much danger from the same source. Dr. Hamilton regarded these cysts as very rare, and from their extreme vascularity, analogous to erectile tissue—operations upon them are dangerous from the excessive number of hœmorrhagic points.

*December 2d,* 1856.—Prof. Hamilton presented, for Dr. C. W. Harvey, a specimen of "'*exostosis*' *of a tooth.*' The patient, a gentleman, had suffered for many years from what had been supposed to be neuralgia, which finally produced insanity. Under these circumstances, he was brought to Dr. Harvey to have a tooth extracted. With great difficulty, and only after applying extraordinary force, he removed this tooth, which was found to be sound, but there is seen attached to it, growing from its roots, near the crown, a round, smooth, solid tumor of bone about the size of a filbert." "The neuralgia immediately ceased, and the patient was soon restored to sanity."

*January* 6, 1857.—Dr. P. H. Strong, from the Committee on "Fistula in Ano in its relations to Pulmonary Consumption," read a long and interesting report, to the effect that operations for the relief and cure of anal fistula were not justifiable but desirable in cases of tubercular diathesis. This conclusion lead to considerable discussion; the views of most of the members were adverse to the decision of the Committee.

At the close of the meeting, the question was raised as to the prophylactic properties of belladonna against Scarletina. Most of those who had tested it thoroughly had abandoned it as entirely useless.

*February 2d,* 1857.—Prof. Hadley presented a specimen of granulated sugar, sold for frosting cake. It consisted of sugar colored with Paris green, an irregular compound of arsenite and acetate of copper. Prof. Hadley had known a whole family poisoned and made very sick by this article, procured from a druggist.

*Tuesday, April 7th,* 1857.—This being the annual meeting, an election was held, resulting in the choice of the following officers: AUSTIN FLINT, President; C. C. WYCKOFF, Vice-President; S. B. HUNT, Secretary; JAMES M. NEWMAN, Treasurer; B. H. LEMON. Librarian; C. L. DAYTON, J. BOARDMAN, A. W. NICHOLS, Primary Board.

The exercises of the evening were concluded by Professors Flint and Rochester, each reading a report on "Epidemic Pharyngitis." The reports were accepted and ordered published. Several other gentlemen made verbal communications upon the same affection.

*August* 4*th,* 1857.—Drs. Rochester and Wyckoff each reported a fatal case of acute rheumatism, occurring in robust middle aged men; both of them were intemperate in their habits. In each instance, apoplectic coma came on suddenly and soon proved mortal.

Dr. Wilcox stated that he had reported a similar case some time ago.

*September* 1*st,* 1857.—Dr. Gay "presented a beautiful specimen of fracture of the neck of the femur, with complete bony union." Prof. Hamilton regarded the specimen as valuable and interesting, but thought the fracture could not have been entirely within the capsule, *because* bony union had taken place. Such an event being, in his opinion, impossible.

Dr. A. W. Nichols reported two instances in which the new nickel and copper cent, coinage of 1857, reputed poisonous, had been swallowed and evacuated by stool, the patients were suffering no inconvenience. He thought the evacuants often employed to dislodge these and other foreign bodies from the alimentary canal, produced the serious results attributed to the object swallowed. Dr. Nichols had been informed by Prof. Hadley that although nickel, as an ore, was found in union with arsenic, that the latter was entirely removed when the nickel was made serviceable for coinage or other use, and was then as inocuous as the ferruginous metals. The only danger was from the copper; but the old copper cent rarely did any harm; and the copper in the new coinage, besides being very much less in amount, was, from its alloy with nickel, less liable to oxidation than when in a pure state.

Prof. Hamilton confirmed Dr. Nichols' statement, giving several analagous instances, and then proceeded to read a report of cases of metallic substances taken into the stomach. Of these we select but a few. An English copper penny, swallowed by a young man 18 years of age. Active cathartics were given; the coin was not dislodged, but fatal hœmorrhage from the bowels ensued. A twenty dollar gold piece, swallowed by Mr.

F. of Buffalo. No inconvenience followed; nor, after the lapse of several years, has it been discharged. A glass door knob, slightly broken; patient eight years old. Castor oil was given; the knob passed on the third day.

Prof. Hamilton gave a long list, selected from various sources, of strange bodies accidently or intentionally swallowed. Those cases that were *let alone* did best.

Most of the members present contributed to the already large record cited, and the mass of evidence was in favor of the "let alone" treatment.

Prof. White next reported an instance of severe rectal hœmorrhage, completely controlled by a *kite tailed tampon.*

Prof. Rochester reported a case of *perforation of the appendix vermiformis,* followed by peritonitis and death. He stated that this was the sixth instance to which he had called the attention of the members of the Association within a few years. He regarded disease of the appendix as one of the most frequent sources of so called idiopathic peritonitis.

Dr. A. Flint, Jr., gave a detailed account of an instance of poisoning by belladonna and syrup of poppies, recently under his observation. He also gave the chief point of three other cases obtained from medical writers. The object being to test the reputed antagonistic properties of opium and belladonna. It did not appear that opium exerted any antidotal influence, but that it prevented dilation of the pupil.

*November* 3*d*, 1857.—Dr. A. Flint, Jr., gave the history of a severe injury of the forearm, by which the middle third of the radius was torn out, the muscles severely lacerated and contused, and the *entire* skin of the forearm separated, except at the wrist and elbow. The integument sloughed away in a few days; but as no important nerves or vessels were injured, it was resolved not to amputate. A good recovery proved the wisdom of this decision.

*December* 1*st*, 1857.—Dr. J. F. Miner read, by invitation, an excellent paper on dissection wounds. He related several remarkable cases which had come under his observation and treatment. Regarded all dead human bodies as poisonous, in greater or less degree, sooner or later—usually the poison acting with greatest virulence very soon after death. Advocated general supporting treatment, with local depletion, especially scarifications and free incisions, in cases where the early manifestations of the disease were found at the point of injury, producing swelling and great pain.

*January* 5*th*, 1857.—Dr. White presented two letters from Dr. Congdon, of Jacksonville, accompanied by two interesting specimens, supposed to be membraneous casts of the cavity of the uterus. The first was obtained from an unmarried woman forty years of age, and was one of several that had been cast off at intervals of five or six days. This, from its frequent renewal, and from its having no connection with the menstrual period, Dr. White regarded as probably adventitious, and similar to the imflammatory fibrous croupy exudation. The second was obtained from a married woman, twenty-six years of age, who had never been pregnant, and who suffered habitually with severe dysmenorrhea, and had thrown of this sac after a violent paroxysm. This Dr. White considered as a true decidua. Both were subjected to microscopic examination by Dr. A. Flint, Jr., in the presence of Dr. White and other profession gentlemen, and both were found identical in structure, and possessing the characteristics of the uterine mucous membrane, viz., tessellated epithelial scales. "No trace of the fibrillation of plastic matter was found in either of the specimens." Remarking upon the microscopic revelations, Dr. White acknowledged that he had been mistaken as to the first specimens, but was glad to find this striking evidence of the facility with which the living membrane of the uterus is thrown off and reproduced.

Dr. Wyckoff gave an account of a case of congenital imperforate rectum; it was entirely relieved by three successive operations, made by Dr. Hamilton and himself.

*February 16th*, 1857.—Dr. White exhibited several growths which he had removed from the cavity of the uterus by Recamier's instrument; they varied in size from that of an apple to a millet seed. Microscopic examination disclosed uterine mucous membrane and areolar tissue. The patient, who had previously suffered from uterine hœmorrhage recovered entirely.

Dr. Rochester then read an account of the illness and death of Dr. Morgan G. Lewis, of Black Rock, a member of the Association. His disease was pyemia.

Dr. A. Flint, Jr., reported, for Dr. Baker, a case of hydrophobia. The patient was a lad 13 years of age. He was bitten, on the first of October, by an animal not thought to be rabid. The wounds bled freely, and healed in a few days. Attention is called to the unusual length of the period of incubation.

Dr. White related a singular occurrence in the case of a patient who was afflicted with erysipelas. He had ordered the muriated tincture of iron for internal administration. By mistake it was applied externally, and with most happy effect.

[The writer takes the liberty of stating, that this fortunate blunder inducted the external use of the tincture of chloride of iron in erysipelas in this city. It has been extensively tested in private and hospital practice for some years, and is esteemed by many practitioners more highly than any other topical remedy.]

*March 2d*, 1858.—Dr. James M. Newman read a most able and elaborate report "On the connection of Albumenuria with the developement of Puerperal Convulsions; and the employment of Chloroform as a medical measure." It was ordered published; and is to be found in Vol. 13, No. 12 of the Buffalo Medical Journal. The paper is replete with detailed and statistical information, and is one of the most creditable of the many valuable contributions to science of its gifted and lamented author.

Prof. Hamilton exhibited a tumor involving the entire parotid gland; every vestige of which he claimed to have removed successfully by excision.

*April 6th*, 1858.—The following officers were elected: Dr. WYCKOFF President; Dr. NEWMAN, Vice-President; Dr. FLINT, Jr., Secretary; Dr. MIXER, Treasurer; Dr. LEMON, Librarian; Drs. HAWLEY, EASTMAN and KING, Primary Board.

Prof. White reported the successful restoration of a uterus after it had been inverted for six months. The lady, a patient of Dr. C. D. Robinson, of Hornellsville, had a speedy and complete recovery.

*May 8th*, 1858.—Dr. Rochester made a report, by appointment, on Tracheotomy in Croup. It concludes as follows: "Tracheotomy from its intrinsic gravity, never to be lightly or precipitately undertaken, affords a probability of recovery when hope from other resources cannot be entertained, and that moment having arrived, it should not be neglected or delayed."

*June*, 1858.—Prof. Austin Flint, as the retiring President of the Association, delivered an annual address on *Conservative Medicine*, taking for his text the maxim of Chomel: "The first object in the mind of the medical practitioner should be, not to do harm; the second object being, to endeavor to do good." The principles inculcated were presented in a clear, precise and convincing form.

*July 6th*, 1858.—The Committee on "The presentation of Medical Accounts," reported in favor of their being sent in *quarterly*, giving at length the arguments for such a conclusion. The report was accepted, adopted, and ordered published in the daily papers of this city.

Prof. White reported a case of parturient laceration of the perineum, involving not only the sphincter ani, but also the lower part of the gut. The patient had no control over faeces or flatulence. He had operated with complete success, adopting essentially the method of Isaac Baker Brown.

Prof. White also reported a case of very severe puerperal eclampsia, speedily relieved by chloroform inhalation. He was called in consultation. Before his arrival, copious bleeding and free purgation had been employed ineffectually. He regarded puerperal eclampsia as sui generis, and neither epileptic or apoplectic.

(TO BE CONCLUDED IN NEXT NUMBER.)

